# Enhancing Surveillance and Diagnostics in Anthrax-Endemic Countries

**DOI:** 10.3201/eid2313.170431

**Published:** 2017-12

**Authors:** Antonio R. Vieira, Johanna S. Salzer, Rita M. Traxler, Katherine A. Hendricks, Melissa E. Kadzik, Chung K. Marston, Cari B. Kolton, Robyn A. Stoddard, Alex R. Hoffmaster, William A. Bower, Henry T. Walke

**Affiliations:** Centers for Disease Control and Prevention, Atlanta, Georgia, USA

**Keywords:** anthrax, surveillance, diagnostics, outbreak response, endemic, global health security, *Bacillus anthracis*, bacteria, zoonoses, enteric infections, respiratory infections, Bangladesh, India, Ghana, Georgia, Ethiopia, bioterrorism and preparedness

## Abstract

Naturally occurring anthrax disproportionately affects the health and economic welfare of poor, rural communities in anthrax-endemic countries. However, many of these countries have limited anthrax prevention and control programs. Effective prevention of anthrax outbreaks among humans is accomplished through routine livestock vaccination programs and prompt response to animal outbreaks. The Centers for Disease Control and Prevention uses a 2-phase framework when providing technical assistance to partners in anthrax-endemic countries. The first phase assesses and identifies areas for improvement in existing human and animal surveillance, laboratory diagnostics, and outbreak response. The second phase provides steps to implement improvements to these areas. We describe examples of implementing this framework in anthrax-endemic countries. These activities are at varying stages of completion; however, the public health impact of these initiatives has been encouraging. The anthrax framework can be extended to other zoonotic diseases to build on these efforts, improve human and animal health, and enhance global health security.

Anthrax is a zoonotic bacterial disease caused by *Bacillus anthracis*, which primarily inhabits herbivorous wildlife and livestock and is usually fatal among these animals. Human infections can result in a high mortality rate if not diagnosed and treated promptly. Humans contract cutaneous anthrax through direct contact of skin or mucosal membranes with *B. anthracis*–infected animals as they are slaughtered or butchered or by handling by-products (*1*–*3*). Ingestion anthrax results from consuming raw or undercooked meat salvaged from infected animals. Inhalation anthrax causes severe disease but rarely occurs naturally in humans; it is acquired through inhaling *B. anthracis* spores aerosolized during contact with or processing of contaminated hides, bones, hair, or wool (*2*). In addition, an incident of injection anthrax, associated with the use of *B. anthracis*‒contaminated heroin, has been reported in Europe (*4*). Among these forms, cutaneous anthrax is the most common, comprising ≈95% of naturally occurring human infections (*3*). In addition to the naturally acquired forms of anthrax, *B. anthracis* is designated as a potential bioweapon, and the risk of acquiring anthrax from laboratory-produced *B. anthracis* spores emphasizes the importance of anthrax surveillance, prevention, and control in anthrax-endemic countries (*5*,*6*).

*B. anthracis* spores can survive in the soil for many years and are distributed worldwide, although the disease is endemic to Africa, Central Asia, the Middle East, and South America (*7*,*8*). The pathogen has a substantial economic and public health impact in countries with limited resources for the development of anthrax control and outbreak response programs. In anthrax-endemic areas, the high mortality rate among livestock can disrupt the subsistence livelihood for families and distress the local agricultural sector. Contact with *B. anthracis*–infected carcasses and by-products routinely leads to human infections and can affect whole communities through the practice of slaughtering sick animals to recoup income or food from the lost animals (*3*,*9*).

The foundation of anthrax control is vaccination of livestock accompanied by rapid outbreak response to limit environmental contamination and human exposure. Animal outbreak response relies heavily on effective surveillance and availability of rapid and reliable laboratory diagnostics. However, countries with underresourced public and veterinary health surveillance programs and laboratory capacity are disproportionately affected by this disease (*8*).

The need to strengthen global capacity to prevent, detect, and respond to public health threats such as anthrax is increasingly being recognized by endemic countries because of their desire to meet requirements under the International Health Regulations 2005 (*10*) and Global Health Security Agenda (GHSA) (*11*). One component of the Centers for Disease Control and Prevention (CDC) GHSA (*12*) activities is an effort to prioritize zoonotic diseases on the basis of criteria selected by the host country (*13*). In the 7 countries where this prioritization has occurred, 4 countries ranked anthrax as 1 of the top 5 zoonotic diseases of major public health concern (*14*). CDC is committed to building anthrax prevention and control capacity in countries prioritizing anthrax as a public health threat or otherwise requesting assistance.

## Framework for Enhancing Anthrax Prevention and Control

CDC’s Bacterial Special Pathogens Branch, part of the Division of High-Consequence Pathogens and Pathology in the National Center for Emerging and Zoonotic Infectious Diseases, works with governments and other international partners to support activities in anthrax-endemic countries that strengthen human and animal anthrax surveillance, enhance laboratory capacity, develop control strategies, and foster collaborative outbreak investigations. The goal of these activities is to reduce anthrax in persons who come in contact with infected animals or their by-products and to reduce the economic effect associated with livestock loss. To achieve these goals, CDC developed a comprehensive framework compiled from multiple published guidelines that outlines a start-to-finish approach to prevent and control anthrax (*15*). The principles and methods described in the framework can be applied in any anthrax-endemic country and can be modified to address specific gaps. 

The framework is subdivided into 2 phases, assessment and implementation (Table), and includes instructions on performing assessments (laboratory, epidemiologic, situational); providing recommendations; and implementing interventions to prevent and control anthrax. Anthrax-endemic countries have already started applying the framework principles and have successfully completed some activities, with some ongoing (Technical Appendix Table).

**Table:** Framework for enhancing anthrax prevention and control in endemic countries

Phase no., title	Activities
Phase I—assessment	Establishment of partnerships
	Surveillance and outbreak response assessment
	Laboratory assessment
	Vaccination assessment
Phase II—implementation	Project identification
	Enhancement of surveillance
	Enhancement of outbreak response capacity
	Enhancement of diagnostic capacity
	Development of targeted studies
	Implementation of prevention and control measures
	Development and dissemination of educational materials

## Phase I—Assessment

### Establishment of Partnerships

CDC collaborates with anthrax-endemic countries that request assistance to improve surveillance and diagnostic capacity. Upon request, CDC identifies key working partners in these countries to initiate collaborations. Cooperative agreements are established with host country partners to strengthen existing and develop new anthrax-related activities and provide technical and financial assistance. The One Health approach, involving both human and animal health stakeholders, is used for the promotion of cross-sectoral integration and coordination of activities for the detection, prevention, and response to endemic anthrax (*16*). CDC works with host country representatives to identify a complete cadre of partners and stakeholders to collaborate on anthrax activities. This cadre might include the ministries of health, agriculture, wildlife, and forestry; national institutes; local universities; hospitals; animal industry; and professional organizations. In addition, international organizations like the World Health Organization, the World Organisation for Animal Health, and the Food and Agriculture Organization of the United Nations are usually identified as partners for in-country activities.

Partnering with CDC country offices and local Field Epidemiology and Laboratory Training Programs (FELTPs) has proven to be an effective mechanism for building collaborations on anthrax. Work in the countries of Georgia, Ghana, India, and Bangladesh was facilitated by CDC country offices and FELTP staff, who provided expertise and assistance with forging relationships with multiple agencies, navigating the political environment, assisting with the outbreak response, and promoting needed and beneficial proposed studies. CDC usually engages with national-level partners; however, anthrax is typically endemic only in focal regions. Thus, control programs are most useful when targeting disease-endemic areas. In countries with >1 disease-endemic region, phased implementation improves the likelihood of success. Factors such as status of surveillance, burden of disease, partners, security, and funding should be considered when selecting a region for initial implementation. Once partnerships and agreements are in place, appropriate assessments of ongoing anthrax-related activities and capacities can be conducted.

### Surveillance and Outbreak Response Assessment

Surveillance assessments progress according to the published protocols for the assessment of disease surveillance and response that are modified to be anthrax-specific and address each country’s needs (*17*,*18*). The initial assessment includes a review of information collected by the surveillance systems for both human and animal anthrax; a report of flow and timeliness; the distribution of anthrax-affected areas throughout the country; the burden of disease (number of outbreaks, illnesses, hospitalizations, deaths, associated costs); and available studies and reports describing anthrax in the country. It is critical to discuss the existing national anthrax surveillance systems’ strengths, weaknesses, and barriers, with a focus on anthrax case definitions, case reporting processes, surveillance data quality, outbreak investigation protocols, and intersectoral collaboration, which provide valuable information on areas for collaboration and project development to enhance anthrax surveillance.

### Laboratory Assessment

Similar to surveillance assessments, laboratory assessments were developed by modifying existing assessment tools and incorporating evaluations for anthrax diagnostic procedures (*2*,*3*). Assessment of laboratory capacity includes identifying existing national, regional, and local laboratories performing anthrax diagnostics. Then, various aspects of the laboratories are evaluated, such as the existing workforce, established diagnostic and logistic capacity, available equipment, facility infrastructure, and waste management. Laboratory assessment findings and the diagnostic capacity that countries request for use within their laboratory system are used to determine the needs for appropriate training, facility improvements, and diagnostic algorithms to ensure the safety of all facility staff.

Numerous diagnostics ranging from basic Gram stains to more specialized culture and molecular diagnostics (e.g., PCR) are available for identifying *B. anthracis.* Each has varied sensitivity and specificity and requires varied technological skills and laboratory resources. Diagnostic capacity varies by country. Most underresourced countries will base their outbreak response on clinical signs and microbiological stains and culture. However, some countries have successfully developed PCR and culture capability to detect and confirm anthrax from clinical specimens. Fortunately, the absence of costly Biosafety Level 3 laboratory facilities is not a limiting factor for safely conducting *B. anthracis* diagnostics. Diagnostic procedures, including molecular diagnostics and bacterial culture, can be safely conducted by trained laboratory staff under Biosafety Level 2 conditions, with handling of infectious material in certified biosafety cabinets (*19*,*20*).

### Vaccination Assessment

Animal vaccination is a vital tool to prevent and control anthrax in animals and, thus, prevent infection in humans (*3*). During vaccination assessments, information is collected on the following: the type of vaccine and bacteria strain used; production site; vaccination coverage of livestock; affordability; and logistics for storage, distribution, and delivery. Although the vaccine is available and subsidized through the government in some countries, vaccine cost is often the livestock owners’ responsibility. Information on vaccination policies and regulations, such as timing, frequency of administration, record keeping, vaccine administration personnel, and minimum age of animals at vaccination, are also collected. Assessment of animal vaccination status is laborious and the information is rarely readily available. Collaboration with vaccine production agencies and commercial partners is essential to obtain these data.

## Phase II—Implementation

### Project Identification

After the assessments, convening multisectoral meetings to discuss priority activities for enhancing anthrax surveillance, diagnostic, and outbreak response capacities and prevention and control measures can ensure a more efficient use of available resources and government ownership of activities. Anthrax stakeholder workshops can help to identify high-risk areas to implement activities and to define and discuss in-country surveillance and laboratory capacity. For example, CDC collaborated with international partners to engage key stakeholders in the country of Georgia through a series of workshops held during 2013–2015 to improve existing systems, promote integration of human and animal anthrax surveillance, and promote rigorous scientific investigations. Similarly, in 2017, CDC organized the Anthrax Surveillance, Prevention, and Control in Ethiopia Meeting, which provided government agencies representing both human and animal health the opportunity for technical discussions of ongoing anthrax activities in Ethiopia, including surveillance, outbreak response, and laboratory diagnostic capacity. The workshop facilitated intersectoral discussions and collaboration to enhance anthrax surveillance and control and identify priority needs for anthrax work in Ethiopia. In addition, CDC assisted partners to coordinate the Bangladesh-India Cooperative Workshop on Anthrax with the goal to strengthen anthrax detection and diagnostics through a coordinated international approach.

### Enhancement of Surveillance, Outbreak Response, and Diagnostic Capacity

During stakeholder meetings, CDC and other partners offer ideas and assistance on activities countries could undertake to enhance their anthrax-related activities, with a focus on improving the areas identified as gaps or weaknesses during assessments. Surveillance can be enhanced by developing an organized reporting system agreed upon by stakeholders, encouraging local (human and animal) health providers to report cases, conducting training courses, providing resources and equipment, and integrating human and animal surveillance data. Anthrax outbreak response can be improved by supporting activities, such as training of response personnel, developing standard operational procedures for joint outbreak investigations, and establishing joint-investigation response teams. Defining clear roles and responsibilities for each agency before an outbreak investigation is critical for an efficient outbreak response. On-site training sessions on outbreak investigations and anthrax diagnostics can target identified gaps and support surveillance of other diseases. In 2016 in Bangledesh, CDC conducted a training on field collection methods for cutaneous lesions and eschars, which included training for sample collection of not only cutaneous anthrax but also other eschar-associated diseases such as poxviruses.

Enhancing outbreak response and surveillance capacity directly affects the country’s ability to detect and contain anthrax outbreaks. In 2012, a national, intersectoral working group was formed in Georgia to investigate a human anthrax outbreak. This group evolved into a One Health surveillance team to improve intersectoral communication and provide more rapid response to anthrax investigations in Georgia. Later, the team promptly identified a human anthrax case in Tbilisi linked to illegally sold meat and traced it back to the seller, preventing a possible outbreak in a dense urban setting (*21*). This team also spurred development of regional rapid response teams to improve surveillance and outbreak response at the local level and developed and disseminated educational materials throughout Georgia. The team affected anthrax control nationwide when they identified animal anthrax reporting issues, which led to targeted interventions in the highest risk districts. These interventions included reinstatement of animal vaccination campaigns in these areas, which resulted in a decline of human anthrax cases (*22*). Furthermore, in 2009 and 2010, CDC assisted the Bangladesh Ministry of Health with its response to multiple anthrax outbreaks, affecting >270 persons. Since this time, CDC has maintained collaborations providing technical support, consultation, and laboratory confirmation for annually occurring anthrax outbreaks throughout Bangladesh (*23*,*24*).

Development of standard operational procedures for specimen collection and transportation, as well as establishment of laboratory diagnostics that are reliable, appropriate, safe, and sustainable, are necessary steps for enhancing anthrax surveillance. Standard diagnostics include microscopy and culture, which are both relatively reliable and sustainable diagnostic techniques. However, biosafety concerns are inherent to culturing bacteria, and identification of culture isolates typically requires confirmation by either PCR or susceptibility to gamma phage, which are not typically available in many anthrax-endemic countries. Increasing a country’s ability to perform molecular diagnostics decreases the turnaround time for specimen processing and diagnostic results (*12*). Thus, CDC encourages the use of molecular methods such as PCR for confirmation at the national reference laboratories. While these diagnostic protocols are being developed and implemented, CDC offers confirmatory testing, such as culture and PCR, for human specimens at the CDC Zoonoses and Select Agent Laboratory in Atlanta, Georgia, USA (*24*). CDC also performs anthrax serologic assays not available in most anthrax-endemic countries, including assays that detect anthrax lethal factor (LF) and anti-protective antigen IgG and measure anthrax lethal toxin neutralization activity levels. CDC has conducted these tests to confirm human outbreaks in Bangladesh; they are specifically useful for identifying outbreaks after implementation of antimicrobial drugs (*23*).

In Bangladesh, CDC used laboratory assessments to identify public health and veterinary laboratories capable of conducting various diagnostic methods and those requiring training and resources to improve methodology, biosafety, and biosecurity to ensure their anthrax diagnostic capabilities. CDC has assisted Bangladesh with diagnostics during anthrax investigations since 2009. A variety of diagnostic methods, including M’Fadyean staining, culture, immunohistochemistry, anti-protective antigen ELISA, toxin neutralization assays, and LF detection by mass spectrometry, were used during outbreaks. This collaborative effort was of great benefit to both CDC and Bangladesh. CDC testing allowed for the first confirmation of human cutaneous anthrax cases in Bangladesh since 1986 and provided CDC invaluable data on the performance of newer tests such as the LF detection test. Unlike patients with inhalation and ingestion anthrax, patients with cutaneous anthrax often do not display systemic illness or bacteremia; thus, the value of testing patient blood for antibodies and LF was unclear. However, these assays were found useful even for diagnosis of cutaneous cases; 18 of 26 probable and confirmed cases of cutaneous anthrax were positive (*23*).

A 2015 assessment of the anthrax diagnostics and laboratory facilities at the Veterinary Services of Ghana, Ghana Health Services, and the Noguchi Memorial Institute for Medical Research in Ghana identified the need for confirmatory diagnostics at the national level. This need was confirmed during discussions with national anthrax surveillance staff, as was the need for a rapid diagnostic test (RDT) to presumptively diagnose animal cases. CDC assisted in training 6 veterinarians from the Veterinary Services of Ghana to use the RDT and collect specimens from animals suspected of dying of anthrax for confirmation and RDT validation. In 2016, the 6 newly trained veterinarians conducted 3 regional training courses, extending capacity to 61 veterinarians. Technology transfer of confirmatory diagnostic methods is planned for 2017‒2018. These efforts to improve diagnostic capacity in Ghana have prompted the development of an electronic notification system for more rapid response to suspected anthrax animal deaths, with the aim to improve surveillance and outbreak response. The use of gamma phage was recently introduced by CDC to veterinary partners in India as a method for diagnostic confirmation of culture-positive isolates in laboratories without PCR capabilities. The use of simple nonmolecular methods, such as infection with gamma phage, has the potential to widen surveillance efforts to bacteriology laboratories where molecular diagnostic capacity is not present.

### Development of Targeted Studies

CDC supports activities aimed at understanding anthrax epidemiology in endemic countries. After a human anthrax outbreak in 2012, CDC collaborated with national and international partners in Georgia to conduct epidemiologic studies to determine the probable sources of environmental and animal exposure. The studies found that humans who had contact with sick or dead animals were at greatest risk of developing anthrax (*25*). CDC also provided technical support for the development and implementation of a matched case–control study to identify risk factors for animal anthrax deaths in Georgia during 2013–2015. This study confirmed the need for regular vaccination of livestock, which was reinstated by the Ministry of Agriculture (*22*). In Bangladesh, CDC are co-investigators with country partners on a study to identify host risk factors associated with cutaneous anthrax infections, aiming to identify vulnerable populations. In this study, risk factors related to animal husbandry practices, socioeconomics, and the geographic distribution of *B. anthracis* are being investigated with the goal to focus future surveillance, prevention, and control strategies in Bangladesh.

### Improving Implementation of Prevention and Control Measures

In Georgia and Bangladesh, surveillance assessments and historical outbreak data were used to target anthrax prevention and control in specific, high-prevalence regions. Spatial modeling of disease distribution can help improve identification and prediction of high-risk areas for anthrax. CDC provided support to partners in Ghana and at the University of Florida (Gainesville, FL, USA) to hold trainings on Geographic Information Systems and spatial modeling for anthrax surveillance. These trainings included 6 Geographic Information Systems webinars with 31 regular participants, followed by 6 days of in-person class to solidify the spatial analytic methods. This same collaboration also resulted in an anthrax predictive risk map for Ghana created by using ecologic niche and random forest modeling. The model is guiding renewed efforts to train medical staff on case identification in high-risk areas and will be used to guide targeted anthrax vaccination campaigns (*26*).

### Development and Dissemination of Educational Materials

Healthcare and community education materials are another aspect of the prevention and control of anthrax. The CDC framework for enhancing anthrax surveillance provides an outline for assessing and implementing anthrax prevention activities in endemic countries. The manual is provided in both English and French and has been distributed to human and animal health partners. In addition, international collaborations have improved communications between CDC and anthrax subject matter experts in anthrax-endemic countries, enabling a more direct, efficient, and mutually beneficial exchange of expertise on anthrax surveillance. Therefore, CDC developed an anthrax toolkit including a series of culturally specific illustrations to communicate anthrax prevention messages. In Cameroon and Mali, these illustrations were used successfully in field manuals for anthrax outbreak control to disseminate a clear One Health message that informs high-risk groups of the health implications of anthrax.

## Impact and Next Steps

Anthrax causes serious public health problems and has high economic significance in affected countries (*9*,*21*). Enhancing surveillance, outbreak response, and diagnostics will prevent anthrax cases in both animals and humans and, thus, will reduce death, illness, and economic losses associated with anthrax. The framework for the control and prevention of anthrax promoting the One Health approach developed by CDC has shown positive public health effects in anthrax-endemic countries (*16*,*27*). The epidemiology of anthrax involves animal, human, and environmental components. Linking human and animal anthrax surveillance and tracing animal outbreaks to their source is imperative for the implementation of effective control measures. Laboratories with enhanced diagnostic capabilities can serve as regional reference facilities, and trained staff can assist with regional anthrax and other zoonotic outbreaks. This work also enhances global health security by supporting the GHSA, which aims to rapidly detect, respond, and control public health emergencies such as anthrax outbreaks.

CDC has provided support for the activities discussed and has seen substantial progress in anthrax prevention and control efforts in each partnering country. Despite the success of the framework activities, additional operational research and other capacity-enhancing activities can and should still be considered. These include assisting countries with building integrated human and animal anthrax surveillance, testing vaccine efficacy, investigating best practices for carcass disposal, and partnering for community education campaigns. In addition, field testing novel, point-of-care diagnostic methods can advance rapid disease detection and biosecurity and enhance diagnostic capacity in endemic areas. Overall, on the basis of positive outcomes from past and ongoing activities, we recommend the continuation of ongoing efforts to support enhancement of anthrax surveillance and diagnostics. The anthrax framework can be adjusted to improve One Health surveillance, prevention, and control of multiple zoonotic diseases in anthrax-endemic countries.

Technical Appendix CDC-supported activities for enhancing anthrax prevention and control in endemic countries.
