# Supplementary material for: Enhancing Surveillance and Diagnostics in Anthrax-Endemic Countries
Source: Emerg Infect Dis. 2017 Dec;23(Suppl 1):S147–53. doi: 10.3201/eid2313.170431 (PMC5711320; doi:10.3201/eid2313.170431)
Supplement: Technical Appendix — CDC-supported activities for enhancing anthrax prevention and control in endemic countries. [file 17-0431-Techapp-s1.pdf]

Article DOI: https://doi.org/10.3201/eid2313.170431

Enhancing Surveillance and Diagnostics in Anthrax-Endemic Countries

Technical Appendix

Technical Appendix Table. CDC-supported activities for enhancing anthrax prevention and control in endemic countries*

Country	Years	Purpose of consultancy	Training/Assistance provided	Participants	Overall impact and public health benefit
Bangladesh	2016–2017	Develop country strategy for prevention and control of anthrax in humans and animals	Stakeholder workshop facilitating intersectoral discussions on enhancing anthrax prevention and control	20 participants, including representatives of Ministry of Health and Ministry of Fisheries and Livestock	Improved coordination and collaboration between human and animal health officials; agreement to focus on same priority endemic areas for active surveillance in next phase of activities
			Review and technical assistance drafting new surveillance protocol and SOPs for sample collection and laboratory diagnostics	Consultation with 5 persons from Ministry of Health	Improved protocol for anthrax surveillance and outbreak response, which should improve identification of anthrax and timeliness of outbreak response activities
		Laboratory diagnostic assistance	Training on PCR diagnostics	11 laboratory scientists and microbiologists trained, including representatives from Ministry of Health and Ministry of Fisheries and Livestock	Improved ability to confirm anthrax outbreaks by human and animal health officials
		Improve sample viability when received at national level laboratory	Training on sample collection and processing	19 field staff responsible for specimen collection from suspect human and animal cases	Improved ability to confirm anthrax outbreaks by human and animal health officials
Bangladesh and India	2016–2017	Training on anthrax surveillance development and laboratory diagnostics	Manipal University in India provided training; logistical and financial support from CDC	6 laboratory scientists and epidemiologists from Bangladesh Ministry of Health	Improved collaboration and training between India and Bangladesh health agencies to address anthrax regionally
		Establishing cross border collaboration and anthrax training	Bangladesh-India cooperative workshop on anthrax	20 veterinary officers, medical officers, scientists, and epidemiologists	Improved collaboration and training between India and Bangladesh health agencies to address anthrax regionally
Ghana	2015–2017	GIS and anthrax laboratory training	6 GIS webinars and 6-d in-person GIS courses	31 participants from Ministry of Health, Ministry of Livestock and Food, Ghana FELTP	Aided in mapping and directing resources for outbreaks
			3-week intensive training on advanced spatial analysis of anthrax	2 participants from Ghana FELTP	Developed models to target livestock vaccination to improve anthrax control in high-risk areas
			Training on sample collection from dead animals with suspected anthrax, and use of RDT	6 veterinarians from Ministry of Livestock and Food and from Ghana FELTP	Train-the-trainer model extended capacity to 61 veterinarians; appropriate sample collection enables anthrax diagnostics; validation of RDT improves timing of diagnosis and response to prevent anthrax in humans and livestock

Country	Years	Purpose of consultancy	Training/Assistance provided	Participants	Overall impact and public health benefit
			Training reference laboratory on PCR and culture diagnostics	3 persons from Noguchi Memorial Institute for Medical Research	Confirmation of anthrax improves sensitivity and specificity of diagnostics
Georgia	2013–2015	Identify source of human anthrax outbreak; continued collaboration on livestock anthrax control	Technical assistance to conduct case–control and cross-sectional studies to determine sources of exposure among human anthrax cases	Collaborative team from NCDC, NFA, LMA, SC-FELTP	Identified sources of exposure; developed communication materials for identified sources; recognized the need for surveillance and disease control improvements
			Anthrax workshop series	Participants from NCDC, NFA, LMA, SC-FELTP	Strengthen interagency relationships; develop plans to improve existing systems; promote integration of human and animal anthrax surveillance; promote rigorous scientific investigations
			Technical assistance to conduct case–control study of livestock anthrax; epidemiology, data quality, and data collection training	Participants from NCDC, NFA, LMA, SC-FELTP	Findings solidified the need for nationally sponsored anthrax vaccination
Ethiopia	2016–2017	Country strategy for prevention and control of anthrax in humans and animals	Stakeholder workshop facilitating intersectoral discussions on enhancing anthrax prevention and control	23 participants from Ministry of Health, Ministry of Livestock and Fisheries, FAO, WHO representing epidemiologists, veterinarians, microbiologists	Development of national anthrax technical working group, with members from the Ethiopian human and animal health agencies

*CDC, Centers for Disease Control and Prevention; FAO, Food and Agriculture Organization; GIS, geographic information system; LMA, Laboratory of the Ministry of Agriculture; NCDC, National Center for Disease Control; NFA, National Food Agency; SC-FELTP, South Caucasus Field Epidemiology and Laboratory Training Program; RDT, rapid diagnostic test; SOP, standard operational procedures; WHO, World Health Organization.
